# Supplementary material for: A Comprehensive Assessment of Ultraviolet-Radiation-Induced Mutations in Flammulina filiformis Using Whole-Genome Resequencing
Source: J Fungi (Basel). 2024 Mar 20;10(3):228. doi: 10.3390/jof10030228 (PMC10971301; doi:10.3390/jof10030228)
Supplement: Supplementary file 1 [file jof-10-00228-s001.zip › Supplementary Material S8/KEGG annotation/out/64381550635650.os/KO/out_map/map05205.html]

KEGG PATHWAY: Proteoglycans in cancer - Reference pathway


|  |  |
| --- | --- |
| **Proteoglycans in cancer - Reference pathway** |  |

[
Pathway menu
| Organism menu
| Pathway entry
| Show description
| User data mapping
]

Many proteoglycans (PGs) in the tumor microenvironment have been shown to be key macromolecules that contribute to biology of various types of cancer including proliferation, adhesion, angiogenesis and metastasis, affecting tumor progress. The four main types of proteoglycans include hyaluronan (HA), which does not occur as a PG but in free form, heparan sulfate proteoglycans (HSPGs), chondroitin sulfate proteoglycans (CSPGs), dematan sulfate proteoglycans (DSPG) and keratan sulfate proteoglycans (KSPGs) [BR:00535]. Among these proteoglycans such as HA, acting with CD44, promotes tumor cell growth and migration, whereas other proteoglycans such as syndecans (-1~-4), glypican (-1, -3) and perlecan may interact with growth factors, cytokines, morphogens and enzymes through HS chains [BR: 00536], also leading to tumor growth and invasion. In contrast, some of the small leucine-rich proteolgycans, such as decorin and lumican, can function as tumor repressors, and modulate the signaling pathways by the interaction of their core proteins and multiple receptors.

|  |  |
| --- | --- |
| Reference pathway | 100% |
